# Extracellular vesicle separation from milk and infant milk formula using acid precipitation and ultracentrifugation

**DOI:** 10.1016/j.xpro.2021.100821

**Published:** 2021-09-15

**Authors:** Anindya Mukhopadhya, Jessie Santoro, Lorraine O’Driscoll

**Affiliations:** 1 School of Pharmacy and Pharmaceutical Sciences & Trinity Biomedical Sciences Institute, Trinity College Dublin and Trinity St. James’s Cancer Institute, Dublin 2, Ireland

**Keywords:** Health Sciences, Molecular Biology, Protein Biochemistry, Protein expression and purification, Biotechnology and bioengineering

## Abstract

Separation of highly enriched extracellular vesicles (EVs) fractions from milk is desirable for quantification, cargo analysis, functional characterization, and investigation as delivery vehicles for nutrients and/or therapeutics. However, a rigorous, reproducible protocol is lacking. This protocol considers a crucial aspect typically overlooked, i.e., that caseins are of similar size to, but more abundant than, EVs in milk. Our protocol combines acid pre-treatment and gradient ultracentrifugation, producing EV-enriched fractions suitable for downstream orthogonal characterization approaches.

For complete details on the use and execution of this protocol, please refer to Mukhopadhya et al. (2021).

## Highlights

• Casein micelles or contaminant-free EVs are best for milk EV characterization
• Acid pre-treatment + gradient ultracentrifugation essential to obtain pure milk EV
• Disrupted and fewer EVs identified in infant milk formula (IMF) than in skim milk
• Notably lower signals of surface markers obtained from IMF EVs than skim milk EVs

## Graphical abstract

**Figure:**
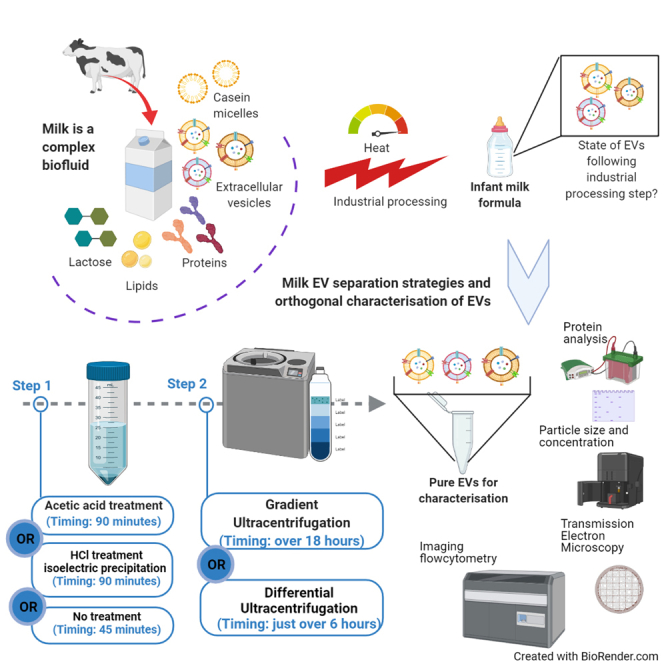

## Before you begin

Here we demonstrate EV separation protocol from commercially available defatted milk (< 1% fat), including bovine skim milk and from infant milk formula. However, this protocol can be broadly applied to milk from different species, including human milk as well as spray-dried milk.

Bovine skim milk (SM, (n=3 independent batches) and infant milk formula (IMF), prepared as recommended by the manufacturer (n=3 independent batches), were purchased from local vendors, as these are the typical sources that would be available to consumers to provide to infants/children. Crucially, a fresh batch of SM and IMF was prepared for each biological repeat experiment.

Each independent batch of SM and IMF was divided into 3 equal aliquots. One was untreated (NT), and the others were treated with either acetic acid (AA) or with HCl for isoelectric precipitation (IP), respectively, to remove abundant casein micelles. After the acid treatment and the three-step filtration, 38 mL of the resultant whey was used for differential ultracentrifugation (DUC) and 2.33 mL of the resultant whey was used for gradient ultracentrifugation (GUC) for separation of highly enriched EV fractions that can be characterized by protein visualization and quantification, nanoparticle tracking analysis, transmission electron microscopy, immunoblotting, and imaging flow cytometry. The GUC approach is more time-consuming (over 18 h) compared to the DUC method (just over 6 h) but, GUC generated the most highly EV-enriched fractions. However, we present both options so the researchers can make an informed choice when applying this to their studies of human milk, bovine milk, infant milk formula (IMF) and, indeed, milk from other species.

All relevant information on the experiment was submitted to EV-TRACK knowledgebase (Van Deun et al., 2017) and the EV-TRACK ID: EV190096 was assigned and the score achieved was 100%.

## Key resources table

**Table:** 

REAGENT or RESOURCE	SOURCE	IDENTIFIER
**Chemicals, peptides, and recombinant proteins**		

Acetic acid	Sigma-Aldrich	Cat# 338826
Hydrochloric acid	Thermo Fisher Scientific	Cat# 1559100
PBS (without calcium or magnesium, pH 7.2)	Sigma Aldrich	Cat# D8537
Iodixanol solution OptiPrep™ (60% (w/v))	Sigma Aldrich	Cat# D1556

**Biological samples**		

Skim milk/ defatted milk (< 1% fat)	Local sources	n/a
Infant milk formula powder	Local sources	n/a

**Other**		

Optima XPN-100 Ultracentrifuge	Beckman Coulter	Cat# A94469
Type 70.1 Ti Fixed-Angle Titanium Rotor	Beckman Coulter	Cat# 342184 (https://www.beckman.com/centrifuges/rotors/fixed-angle/342184)
SW Type 32 Ti swinging bucket rotor	Beckman Coulter	Cat# 369650 (https://www.beckman.com/centrifuges/rotors/swinging-bucket/369650)
17 mL polyallomer tube	Beckman Coulter	Cat# 344061
38 mL open-top polypropylene tubes	Beckman Coulter	Cat# 326823
pH meter	Mettler Toledo, FiveEasy Standard pH Meter Line	Cat# 30266627
Sorvall ST 8 R centrifuge	Thermo Fisher Scientific	Cat# 75007205
Pal-Ri Refractometer	ATAGO	Cat# 3850
Whatman^TM^ Grade 1 filter paper (diameter 150 mm)	GE Healthcare Life Sciences	Cat# 1201
0.45-μm membrane syringe filters	Thermo Fisher Scientific	Cat# 15216869
0.22-μm membrane syringe filters	Thermo Fisher Scientific	Cat# 15206869
Disposable needles, 18-gauge, 1.5in. long, w/polypropylene luer-fitting	BD Microlance	Cat# 304622
Disposable syringe, 10 mL, luer-lock, sterilized	VWR	Cat# 613-2008

## Step-by-step method details

### Preparation of untreated milk samples

**Timing: 45 min**

**Figure 1:**
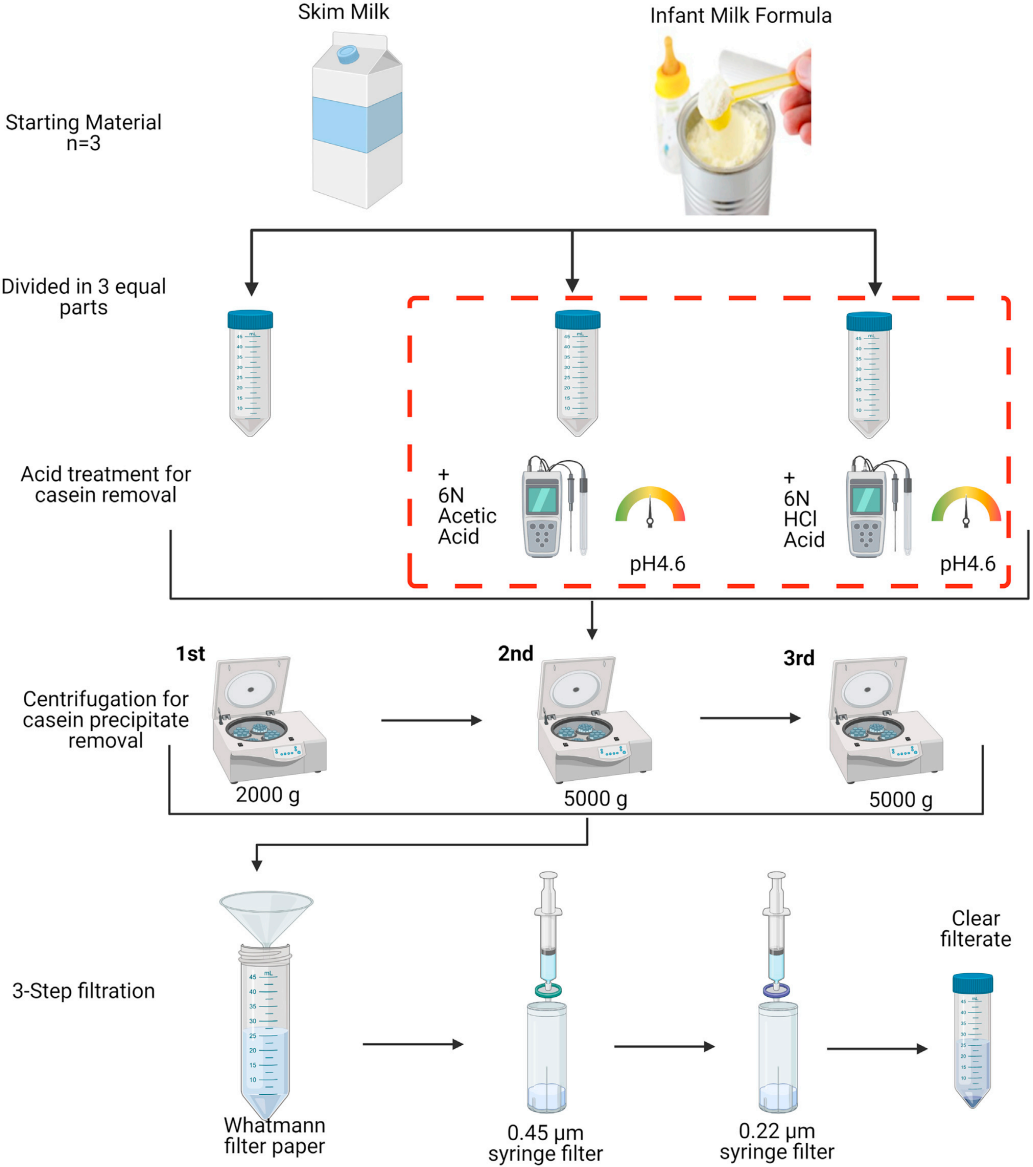
Acid treatment for removal on non-EV related proteins, in particular caseins This figure outlines the two treatments performed on milk to remove non-EV related proteins, especially caseins as they can act as contaminant proteins due to their abundance and overlapping sizes. Both SM and IMF were either left untreated or treated with 6 N acetic acid (AA) or Hydrochloric acid (HCl). Post-acid treatment, for removal of the precipitates, samples were centrifuged on a benchtop centrifuge and further used in a 3-step filtration process, as shown in the figure. Of note, the non-treated samples were filtered only using the filter paper as filtration using syringe filters was not possible due to clogging of filters by milk proteins.

This part of the protocol is presented in Figure 1.

***Note:*** Commercially available SM and powdered IMF were used in this study. Fresh SM and freshly prepared IMF samples were always used for EV separation steps. The preparation of IMF from its powder form was performed exactly as recommended by its manufacturer, to replicate the product that would be prepared to feed to infants. Specifically, in the example shown in this paper, 4.4 g of IMF powder was mixed with 30 mL of water that had been freshly boiled and then allowed to cool naturally. When prepared following the instruction on the container, the IMF was described by its manufacturer as a ‘nutritionally complete’ breast milk substitute with 0.7 mg per 100 mL feed iron content.

**CRITICAL:** Care should be taken when dissolving the IMF; only warm, not boiling, filtered water should be used for reconstituting the powder. We suggest following the manufacturer's recommendation when reconstituting any form of spray-dried IMF/milk to successfully separate EVs -as they would exist- from the source. Sterile tubes and filtered deionised water should be used for all steps.

1. For the untreated samples, centrifuge 50 mL of SM or IMF at 2,000 *g* for 20 min at 4°C. Carefully collect the supernatant and discard the pellet containing any fat globules or debris. In case non-defatted milk is used as a starting material, centrifuge milk at 6,000 *g* for 10 min at 4°C to remove fats and then proceed with the supernatant and centrifuge at 2,000 *g* for 20 min at 4°C.
2. Pass this supernatant through Whatman^TM^ Grade 1 filter paper (diameter 150 mm) using a glass funnel. Fold the filter paper in half and, then again, fold it to form a 90° center angle. Place the folded filter paper on one side in the funnel and open one layer on the other side to form a funnel. Carefully pour the supernatant and collect the filtrate in a fresh tube at the bottom of the funnel (Figure 4A).
3. The filtrate obtained from untreated SM is designated as SM_NT and the filtrate from untreated IMF is designated as IMF_NT.

### Preparation of acetic acid-treated milk samples

**Timing: 90 min**

**Figure 2:**
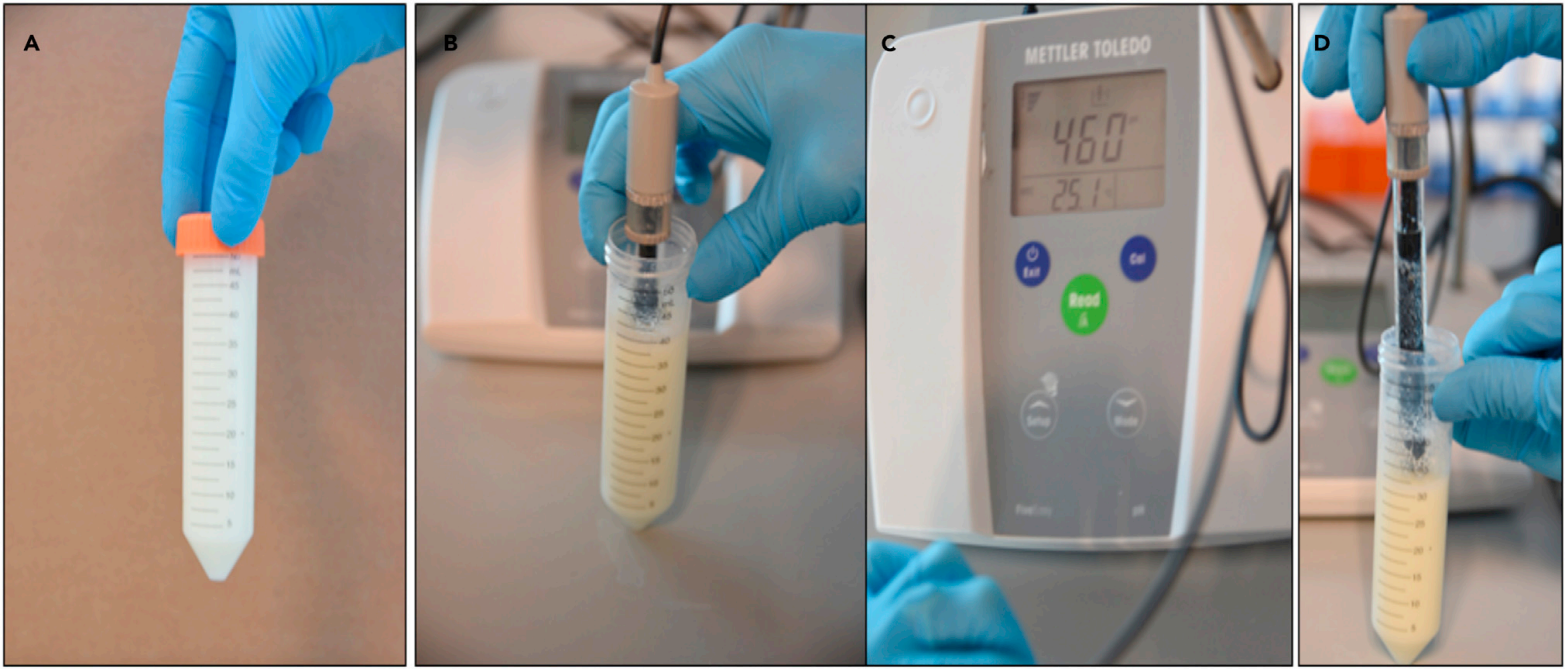
Isoelectric precipitation of casein micelles (A) 50 mL of skim milk harvested from cell debris. (B) Adjustment of the pH to 4.6 (isoelectric point) using 6 N HCl (left) (C) Measurement of the desired pH using a pH meter (right). (D) Milk after adjustment of the pH

**Figure 3:**
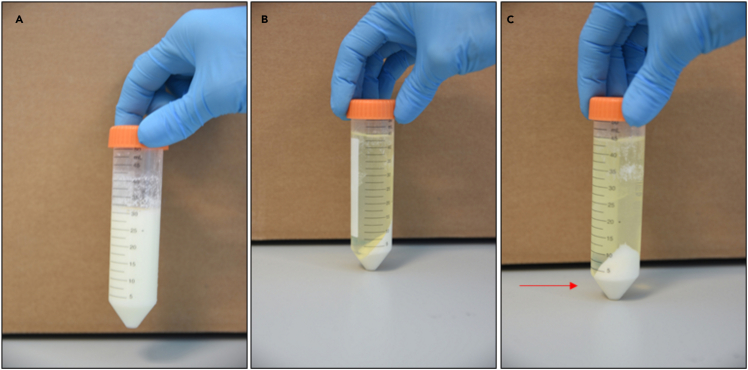
Casein micelles precipitation and whey separation (A) Milk at pH 4.6 (left) versus milk after 20 min centrifugation at 5000 g (right) (B) Caseins are precipitated after centrifugation and separated from milk whey (C) The precipitated casein proteins (arrow) are pelleted

**Figure 4:**
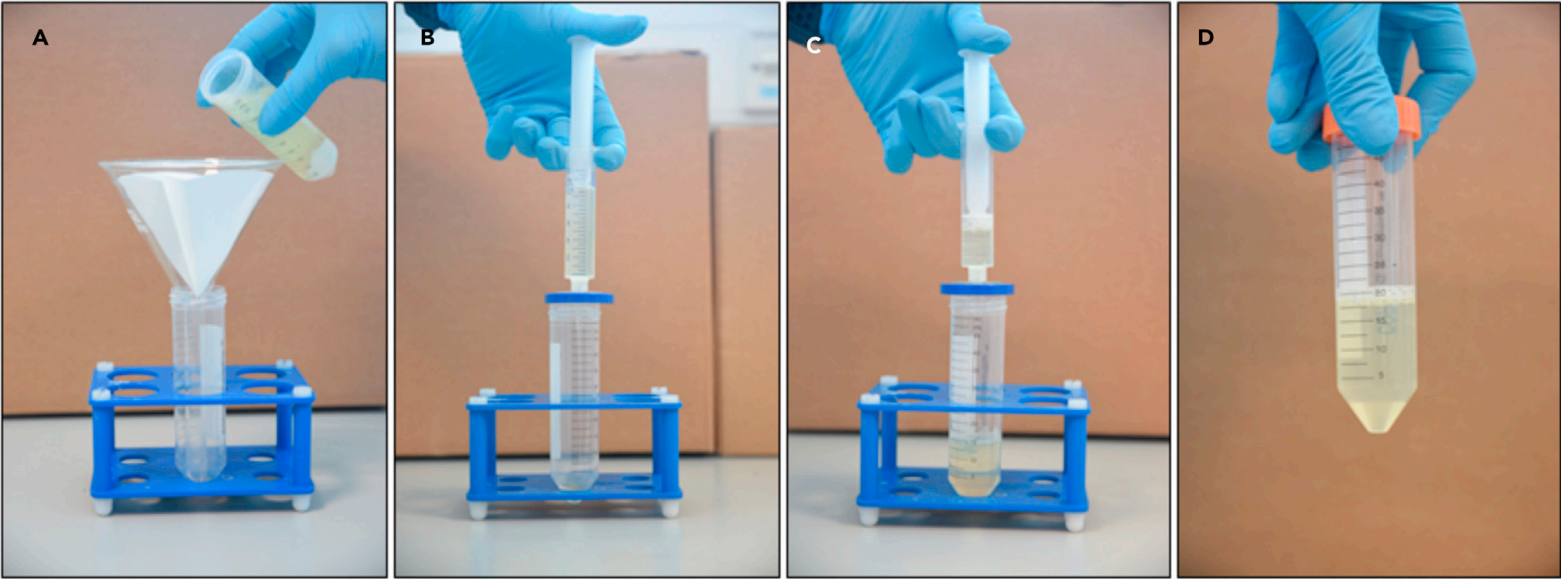
Three-step filtration of milk whey (A) Milk whey separated from caseins and filtrated using Whatman filter paper. (B) 0.45 μm syringe filter is used to filtrate milk whey. (C) 0.2 μm syringe is used to achieve total remotion of casein residues. (D) 20 mL of filtrated milk whey is transferred in a falcon tube

This part of the protocol is presented in Figures 1, 2, 3, and 4.

The acetic acid treatment of SM and IMF was performed based on a protocol published by Somiya et al. (2018) but with extra filtration steps as described in steps 4–6 below.

**CRITICAL:** For use of acid in this step, perform the task under a fume hood.

4. To adjust the pH of milk to 4.6, 6 N acetic acid was used. A standard pH meter (Mettler Toledo) was used to measure the pH. Acid is added to milk drop-by-drop, making sure the pH is changed in a controlled manner while stirring the milk sample continuously (Figure 2).
5. Once the pH is adjusted to 4.6, the sample is left for 10 min at room temperature (20°C–22°C), followed by centrifugation (Sorvall ST 8 R benchtop centrifuge) at 5,000 *g* for 30 min at 4°C (Figure 3).
6. A 3-step filtration, using Whatman^TM^ Grade 1 filter paper (diameter 150 mm), 0.45-μm and 0.22-μm membrane syringe filters, was performed (Figure 4). The filtrate from SM is designated as SM_AA and that from IMF is designated as IMF_AA.

**CAUTION**: Store the resultant whey at 4°C until further use, do not freeze.

### Preparation of isoelectric precipitated milk samples

**Timing: 90 min**

This part of the protocol is presented in Figures 1, 2, 3, and 4.

Isoelectric precipitation (IP) was performed on SM and IMF based on the protocol published by Yamauchi et al.(2018) with modifications as described in steps 7–9 below.

**CRITICAL:** For use of acid in this step, perform the task under a fume hood.

7. Dilute the SM and IMF samples with an equal volume of distilled water and adjust the pH to 4.6 with 6 N HCl to precipitate caseins, following the details mentioned in point 4 above (Figure 2).
8. The pH adjusted milk is placed on ice for 10 min, followed by centrifugation at 5,000 *g* for 30 min at 4°C. Whey is filtered as described above (Figure 4).
9. The filtrate from SM is designated as SM_IP and that from IMF is designated as IMF_IP (Figure 4).

**CAUTION**: Store the resultant whey at 4°C until further use, do not freeze.

### Separation of EVs by ultracentrifugation

**Figure 5:**
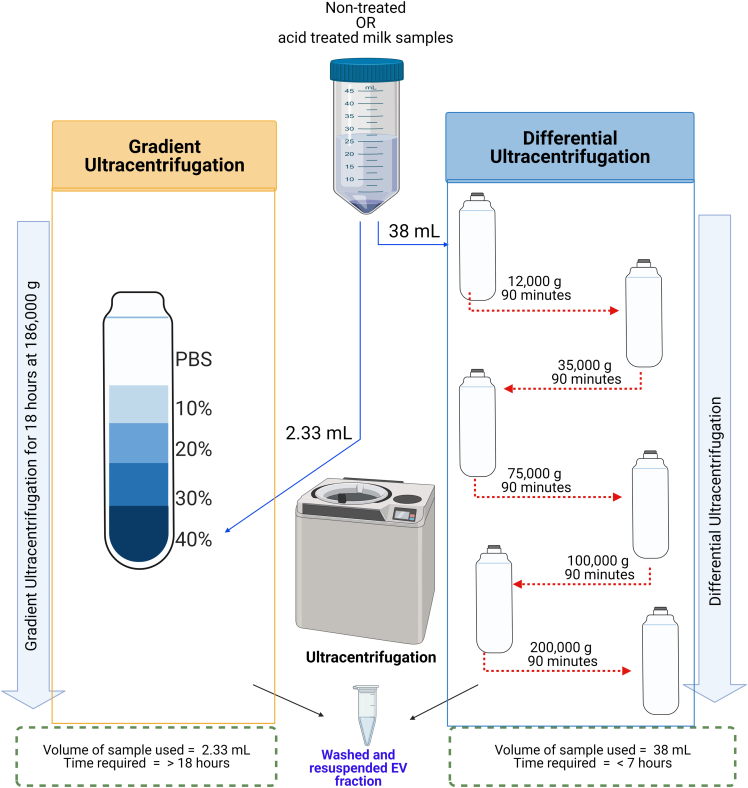
Ultracentrifugation steps to separate EVs This figure outlines the ultracentrifugation techniques used for each sample as well as outlines the volumes required and the time required to carry out the process. Untreated, AA and IP treated milk samples were further subjected to either Gradient ultracentrifugation (GUC) or Differential ultracentrifugation (DUC). GUC required 2.33 mL of the sample that was mixed with Optiprep to obtain a 40% solution. This was layered at the bottom of a 17 mL tube followed by 30%, 20% and 10% solution of Optiprep solution in PBS. PBS was layered on the top of the tube and the tube was ultracentrifuged at 186,000 *g* for 18 h at 4°C. On the other hand, for DUC, 38 mL of sample was loaded in a polypropylene tube that was centrifuged at 12,000 *g*, 35,000 *g*, 75,000 *g*, 100,000 *g* and 200,000 *g* for 90 min each at 4°C. The final pellet was collected and stored for further use.

The outline of the ultracentrifugation steps is graphically presented in Figure 5.

**CRITICAL:** The following steps include the use of ultracentrifugation and so proper care related to the use of an ultracentrifuge should be taken. The rotors are required to be stored at 4°C, as per the manufacturer’s suggestion. After filling up the tubes, a microbalance was used to balance the tubes. All the tubes were balanced to achieve the same weight ± 0.01 g/mL.

### Gradient ultracentrifugation

**Timing: 20 h**

**Figure 6:**
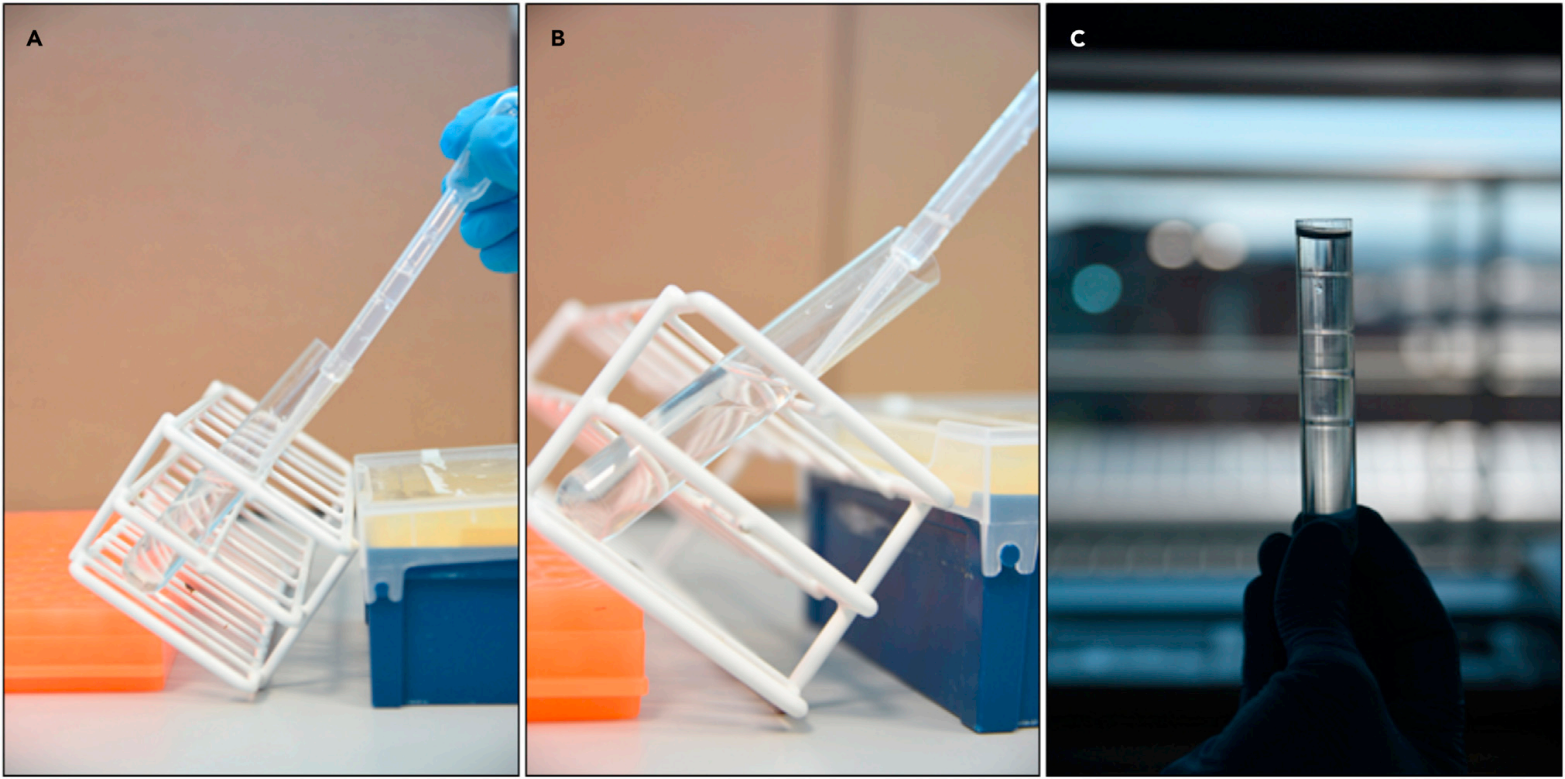
Preparation of the Optiprep density gradient (A) 2.5 mL of 30% Optiprep is carefully loaded on top of a 40% layer (60% Optiprep + 2.33 mL milk whey. (B) Ultraclear centrifuge tube is gently disposed of at approximately 70° angle to facilitate the loading of the layers. (C) The density gradient is prepared.

10. Gradient ultracentrifugation (GUC) using an SW Type 32.1 Ti rotor in Optima XPN-100 Ultracentrifuge (Beckman Coulter, Brea, CA, USA) is performed using iodixanol solution OptiPrep™ (60% (w/v)) following the bottom-up technique.
11. A 40% (w/v) bottom layer is made by diluting 60% Optiprep solution with each sample type (4.67 mL Optiprep + 2.33 mL sample = 7 mL).
12. Transfer the 7 mL of the solution into the 17 mL polypropylene tube carefully, placing the tube in an upright position.
13. After the transfer of the solution, place the tube at an approximately 70° angle to form the discontinuous gradient layers (Figure 6).

**CRITICAL:** The formation of the discontinuous gradient layering is done carefully without disturbing the layers formed inside the tube. For this, the respective iodixanol solution was added drop by drop, using a pipette.

14. Layer the discontinuous gradient with 30% (w/v), 20% (w/v), 10% (w/v) and 5% (w/v) solutions of iodixanol (from bottom to top), that have been made by diluting a stock solution of OptiPrep (60% (w/v)) with sterile PBS (Figure 6).

**CRITICAL:** If multiple gradient tubes are being prepared, store the prepared tubes either at 4°C or on ice until the other tubes are ready.

15. After the gradient tube preparation, carefully transfer the tubes into their respective rotor holders.
16. The holders are weighed to balance all the tubes to the same weight ± 0.01 g/mL using a microbalance.

**CRITICAL:** The parameters set for the ultracentrifugation step should be followed. Any changes to the step may lead to inaccurate separation based on density. Also, care should be taken so as the acceleration and deceleration (before and after centrifugation, respectively) of the ultracentrifuge do not occur abruptly, which may lead to the disruption of the separated layers.

17. Carefully place the holders in the pre-cooled rotor and set the ultracentrifuge with the following parameters on the software:

**Table:** 

Type 70.1 Ti fixed angle rotor	
Parameter	Value
Speed	186,000 g
Time	18 h
Temperature	4°C
Acceleration	0
Deceleration	0

18. Ultracentrifugation is performed following the above-mentioned parameters.

**CRITICAL:** After the centrifugation step, 1 mL fractions are collected; a total of 17 tubes corresponding to 17 mL volume of the polypropylene tubes. The collection of the 1 mL fractions is carried out carefully without disrupting the layers formed. The collection of the fractions should always be done from top to bottom and the sample should carefully be pipetted out from the centre of the liquid surface using a P1000 pipette.

19. Post 18 h of centrifugation, 1 mL fractions are collected, starting from the top to bottom.

### Refractive index determination and pooling based on densities

**Timing: 60 min**

20. Using a hand-held Pal-Ri Refractometer, the refractive index (RI) of each fraction is determined.
21. First, the refractometer is zeroed using 300 μL of distilled water.
22. Once calibrated, 300 μL of each fraction is placed on the refractometer for each reading.
23. Resolution is displayed to four decimal places.
24. Based on the measured RI, the following equation is used to calculate the density:

Density = 3.3411 × RI – 3.4584

25. Following previously published research (Admyre et al., 2007; Zonneveld et al., 2014), and based on the calculated densities, fractions are pooled as follows:
  a. pool 1 (fractions with density ≤ 1.00 g/mL)
  b. pool 2 (fractions with densities between 1.10 to 1.20 g/mL)
  c. pool 3 (fractions with density ≥ 1.20 g/mL)

**CRITICAL:** After the pooling of the fractions based on densities, the iodixanol solution is washed by adding a PBS plus centrifugation step. In separate polypropylene tubes, the pooled fractions and sterile PBS are added to bring the volume up to 38 mL. The tubes are balanced to a similar weight ± 0.01 g/mL. The balanced tubes are placed in a pre-cooled SW Type 32 Ti swinging bucket rotor and ultracentrifugation is performed using the following parameters:

**Table:** 

SW type 32 Ti swinging bucket rotor	
Parameter	Value
Speed	110,000 g
Time	90 m
Temperature	4°C
Acceleration	0
Deceleration	0

26. After centrifugation, all the liquid is carefully pipetted out and discarded and the viscous pellet at the bottom is resuspended with 1 mL of sterile PBS.

**CAUTION**: Store the resultant EV-enriched suspension at −80°C until further use.

### Differential ultracentrifugation

**Timing: 8 h**

**CRITICAL:** The following steps include the use of ultracentrifugation and so proper care related to the use of ultracentrifuge should be taken. The rotors are required to be stored at 4°C, as per the manufacturer’s suggestion. After filling up the tubes, a microbalance is used to balance the tubes. All the tubes are balanced to achieve the same weight ± 0.01 g/mL.

Differential ultracentrifugation (DUC) separation of EVs from the untreated or treated SM and IMF samples is performed based on a protocol previously published by Benmoussa et al. (2017), with modifications as described below in steps 27–29.

27. Samples are filled into 38 mL open-top polypropylene tubes and balanced to their weight (± 0.01 g/mL difference) before placing into the rotor specific holder.
28. The weighed tubes are carefully placed into the pre-cooled SW Type 32 Ti swinging bucket rotor and ultracentrifugation is performed using the following parameters:

**Table:** 

SW type 32 Ti swinging bucket rotor	
Parameter	Value
Speed	12,000 g, 35,000 g, 75,000 g, 100,000 g and 200,000 g
Time	75 m for each speed
Temperature	4°C

29. After each centrifugation step, the pellet (denoted as 12 K, 35 K, 75 K, 100 K or 200 K, respectively) is collected, resuspended in 1 mL PBS and stored at −80°C for potential further analysis, whereas the supernatant is poured in a fresh tube and centrifuged at the next speed.

**CAUTION**: Store the resultant pellets at −80°C until further use.

## Expected outcomes

While previous studies have attempted to separate EVs from milk, in our published study (Mukhopadhya et al., 2021) we established that using ultracentrifugation alone is not adequate to remove most casein micelles from milk and that a pre-treatment is required to separate the most enriched EVs possible. Studies such as those described by Somiya et al. (2018) and Yamauchi et al. (2018) have reported the use of acetic acid or HCl, respectively. However, casein removal was not achieved. Additionally, Benmoussa et al. (2017) used a 4 step differential ultracentrifugation method, with 100,000 *g* as the final EV separation step, which might be a limitation of this study. Milk EVs are potentially separated by centrifugation at 200,000 *g*, as previously reported by Somiya et al. (2018). Hence, to overcome this possible limitation, we added the 200,000 *g* ultracentrifugation step to our milk EV separation protocol. We reported that in our protocol, that includes IP pre-treatment and then GUC, while requiring a longer time than the DUC protocol, requires comparatively less starting material to achieve comparable amounts of EVs and the EVs separated can be successfully characterized.

Following the acid pre-treatment plus GUC protocol described here, the EV enriched fractions were separated from SM and IMF samples and were subjected to protein characterization, particle characterization, transmission electron microscopy and imaging flow cytometry analysis, comparing the status of EVs from IMF to those from its starting material SM. The details of the characterization steps and the outcomes can be accessed in our publication (Mukhopadhya et al., 2021).

**Figure 7:**
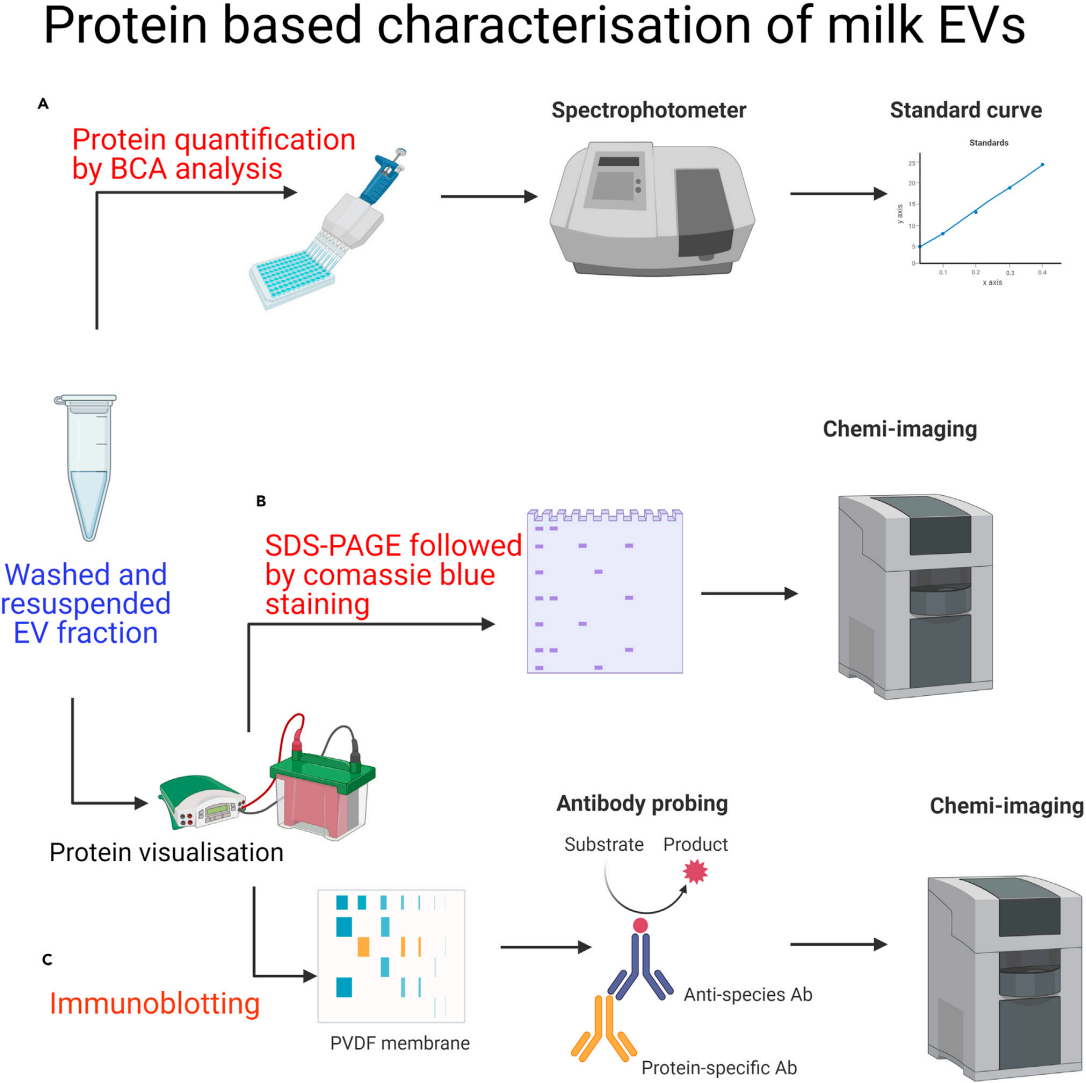
Graphical representation of protein characterization of skim milk and infant milk formula EVs by BCA, SDS-PAGE and Immunoblotting EV enriched fractions from skim milk (SM) and infant milk formula (IMF) post gradient ultracentrifugation (GUC) and differential ultracentrifugation (DUC) were analyzed for protein concentrations using (A) bicinchoninic acid (BCA), (B) SDS-PAGE and (C) immunoblotting

**Figure 8:**
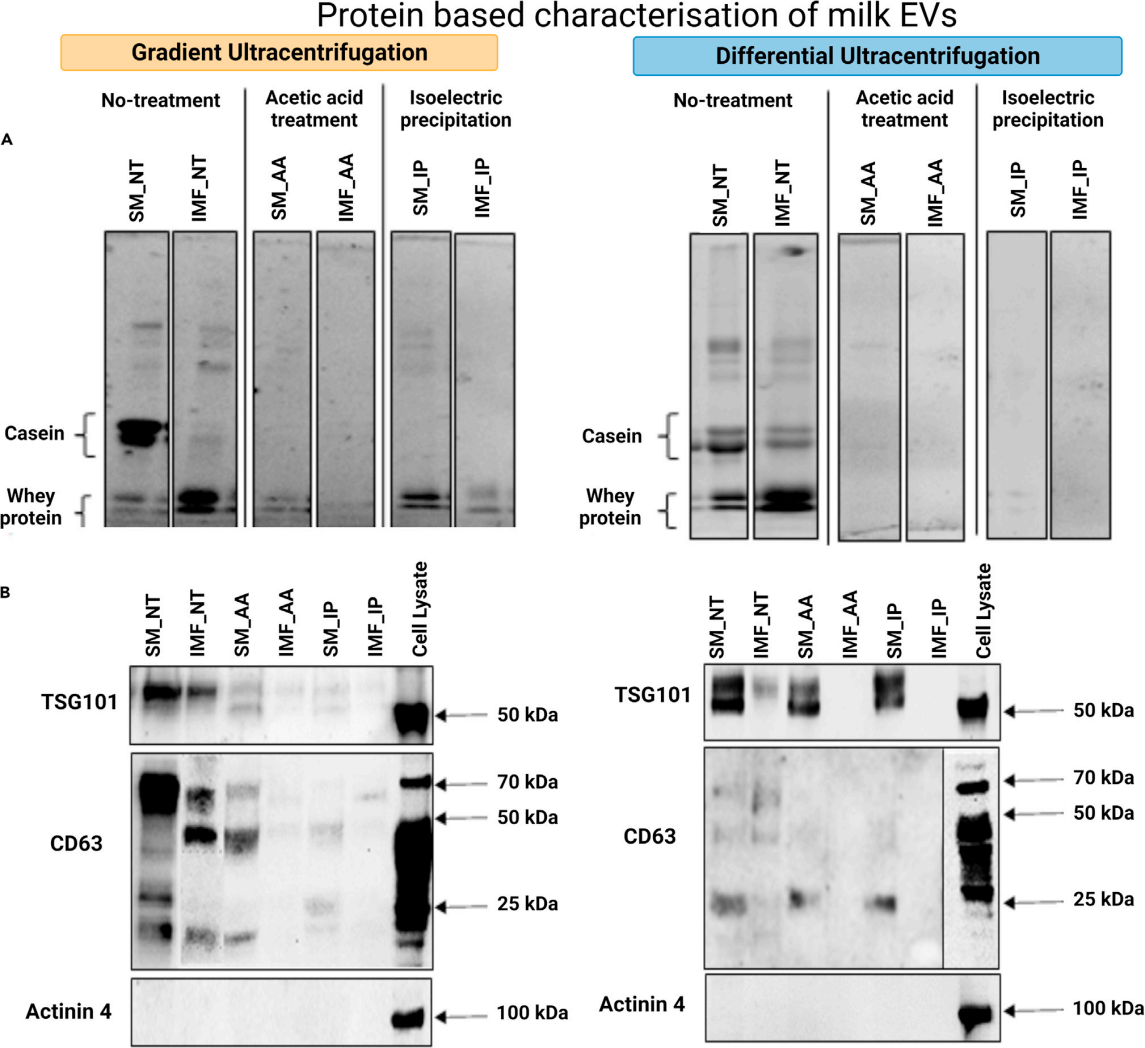
Representative blots obtained by SDS-PAGE and Immunoblotting (A) Milk proteins were visualized on 10% acrylamide gels. Efforts to remove milk proteins were performed by acetic acid (AA) and isoelectric precipitation (IP) and the resulting whey was used for either GUC or DUC. Untreated milk (NT) was used as a control to compare the removal of milk proteins by AA or IP pre-treatment. (B) Immunoblotting of SM and infant milk formula (IMF) EVs separated using GUC and DUC and their densitometric analysis. An equal amount of protein (35 μg) was loaded for all the samples and analyzed for TSG101, CD63 and Actinin 4. Figure reprinted with permission from Mukhopadhya et al. (2021).

**Protein characterization:** Protein characterization was performed by BCA, SDS-PAGE and immunoblotting, as presented in Figure 7. The total protein separation by polyacrylamide gel showed that the major groups of milk proteins, including caseins and whey proteins, were present post-GUC at high levels in the SM_NT samples and to a lesser extent in IMF_NT samples (Figure 8A), while pre-treatment with AA or IP quite efficiently removed caseins. Post-DUC, the SM_NT and IMF_NT had strong bands representing caseins and whey proteins and AA and IP pre-treatment efficiently removed these proteins. Therefore, AA and IP samples, especially those generated by GUC were termed as EV enriched fractions, whereas NT or those generated by DUC were termed as crude samples.

In line with MISEV2018 guidelines (Thery et al., 2018), immunoblotting was performed on lysed samples for two EV positive markers, TSG101 and CD63, and a negative marker Actinin 4 and a representative blot presented in Figure 8B. The GUC generated SM_NT, SM_AA and SM_IP samples were typically positive for both TSG101 and CD63, but not Actinin 4. Although equal quantities of protein were loaded, TSG101 and CD63 were always at higher levels in SM-derived compared to IMF-derived samples and Actinin 4 was undetected in all EV samples. In post-DUC samples, no signal for Actinin 4 was observed for either SM or IMF. Again, SM_NT, SM_AA and SM_IP samples were positive for both TSG101 and CD63. IMF_NT samples compared to SM_NT had faint TSG101 and CD63 signals. Unlike SM_AA and SM_IP samples, neither IMF_AA nor IMF_IP samples showed bands for either TSG101 or CD63.

**Figure 9:**
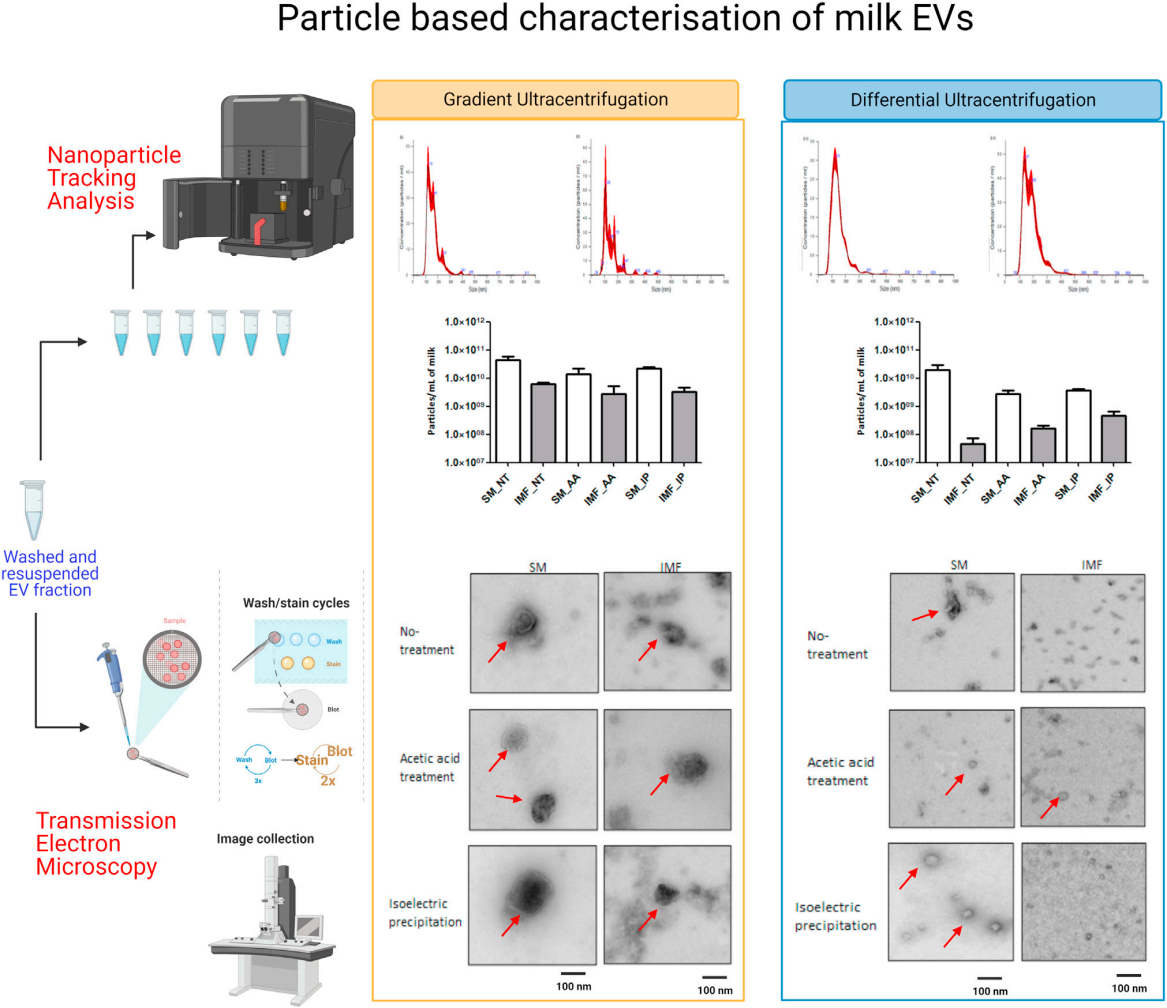
Particle characterization and visualization of skim milk and infant milk formula EVs by nanoparticle tracking analysis and transmission electron microscopy EV fractions generated post ultracentrifugation (GUC), and differential ultracentrifugation (DUC) were analyzed using a NanoSight NS300 analyzer (top panel). The EV enriched fractions were diluted accordingly, injected into the analyzer and the NTA 3.1.54 software was used to capture and analyze the EV size and particle concentration of the sample. Representative transmission electron microscope (TEM) images from GUC and DUC samples (bottom panel). Red arrows point towards vesicular structures in the sample. Scale bar=100 nm. Figure reprinted with permission from Mukhopadhya et al. (2021).

**Particle characterization:** The size and concentration of EVs separated from SM and IMF samples were analyzed by NTA and observed data presented in Figure 9A and mean of size and yield in Table 1. Considering size distribution, post-GUC particles from the SM (SM_NT, SM_AA and SM_IP) samples were 156.13±5.87 nm and from IMF (IMF_NT, IMF_AA and IMF_IP) samples were 159.90±12.96 nm. Post-DUC of SM (SM_NT_200 K, SM_AA_200 K and SM_IP_200 K) were 159.10±5.27 nm and IMF (IMF_NT_200 K, IMF_AA_200 K and IMF_IP_200 K) were 175.73±8.44 nm. No significant differences were observed between the EV sizes of SM and IMF, separated by GUC or DUC.

**Table 1:** Yields of EVs separated from skim milk (SM) and infant milk formula (IMF) by gradient ultracentrifugation (GUC) or differential ultracentrifugation (DUC), as estimated by Nanoparticle Tracking Analysis

i) Gradient ultracentrifugation					
Size (in nm)	Skim milk		Infant milk formula		p-value
	Mean	Mode	Mean	Mode	
Non-treated (NT)	147.00 ± 8.39	113.60 ± 10.34	166.15 ± 2.75	140.45 ± 1.25	Ns
Acetic acid-treated (AA)	159.83 ± 5.55	135.46 ± 5.28	180.16 ± 16.77	133.53 ± 16.22	Ns
Isoelectric precipitation (IP)	162.90 ± 13.51	141.03 ± 11.93	152.40 ± 45.10	109.50 ± 18.80	Ns

Concentration (Particles/mL starting material)	Skim milk	Infant milk formula	
Non-treated (NT)	4.35 × 1010 ± 1.40 × 1010	6.13 × 1009 ± 8.17 × 1008	0.05
Acetic acid-treated (AA)	1.39 × 1010 ± 8.25 × 1009	2.82 × 1009 ± 2.33 × 1009	Ns
Isoelectric precipitation (IP)	2.16 × 1010 ± 2.47 × 1009	3.22 × 1009 ± 1.45 × 1009	0.01

ii) Differential Ultracentrifugation					
Size (in nm)	Skim milk		Infant milk formula		p-value
	Mean	Mode	Mean	Mode	
Non-treated (NT_200 K)	173.60 ± 29.20	123.40 ± 60.00	180.40 ± 7.50	152.50 ± 50.10	Ns
Acetic acid-treated (AA_200 K)	150.10 ± 16.40	146.90 ± 47.33	149.28 ± 18.37	137.30 ± 68.22	Ns
Isoelectric precipitation (IP_200 K)	149.23 ± 23.50	130.81 ± 46.11	184.19 ± 15.63	117.10 ± 67.70	Ns

Concentration (Particles/mL of starting material)	Skim milk	Infant milk formula	
Non-treated (NT_200 K)	1.97 × 1010 ± 8.90 × 1009	4.65 × 1007 ± 2.50 × 1007	0.04
Acetic acid-treated (AA_200 K)	2.78 × 1009 ± 1.00 × 1009	1.69 × 1008 ± 3.30 × 1007	0.03
Isoelectric precipitation (IP_200 K)	3.65 × 1009 ± 5.43 × 1008	4.66 × 1008 ± 2.02 × 1008	0.01

ns, not significant

Table reprinted with permission from Mukhopadhya et al., 2021.

As presented in Table 1 (i), post-GUC, the concentration of EVs in SM_NT samples was higher (7.10-fold; P=0.05) compared with IMF_NT samples. Similarly, the EV concentration of SM_IP samples was significantly higher (6.71-fold; P=0.01) when compared with IMF_IP samples. In turn, the concentration in SM_AA samples was substantially higher (4.93-fold) than in IMF_AA, although not significantly different (P=0.26) as a result of quite large differences in biological repeat SM_AA values (i.e., a large error bar).

Similarly, post-DUC -as presented in Table 1 (ii)- the EV/particle concentration in SM_NT_200 K samples was significantly higher (423.66-fold; P=0.04) than in IMF_NT_200 K. Likewise, the EV/particle concentration in SM_AA_200 K samples was 16.45-fold higher (P=0.03) compared to that in IMF_AA_200 K and the SM_IP_200 K samples had 7.83-fold more particles (P=0.01) when compared to the IMF_IP_200 K samples.

Complementary information was obtained by TEM and presented in Figure 9B. Following GUC separation, the SM EVs (SM_NT, SM_AA and SM_IP) appeared intact with limited background debris. IMF-derived EVs appeared more diffuse with less smooth surfaces, and with much more debris in the background. Post-DUC, SM EVs samples (SM_NT, SM_AA and SM_IP) multiple smaller vesicles were observed, although sometimes in clumps (arrows, Figure 9B). The IMF samples (IMF_NT, IMF_AA and IMF_IP) had very few EV-like structures, but much more background debris.

**Figure 10:**
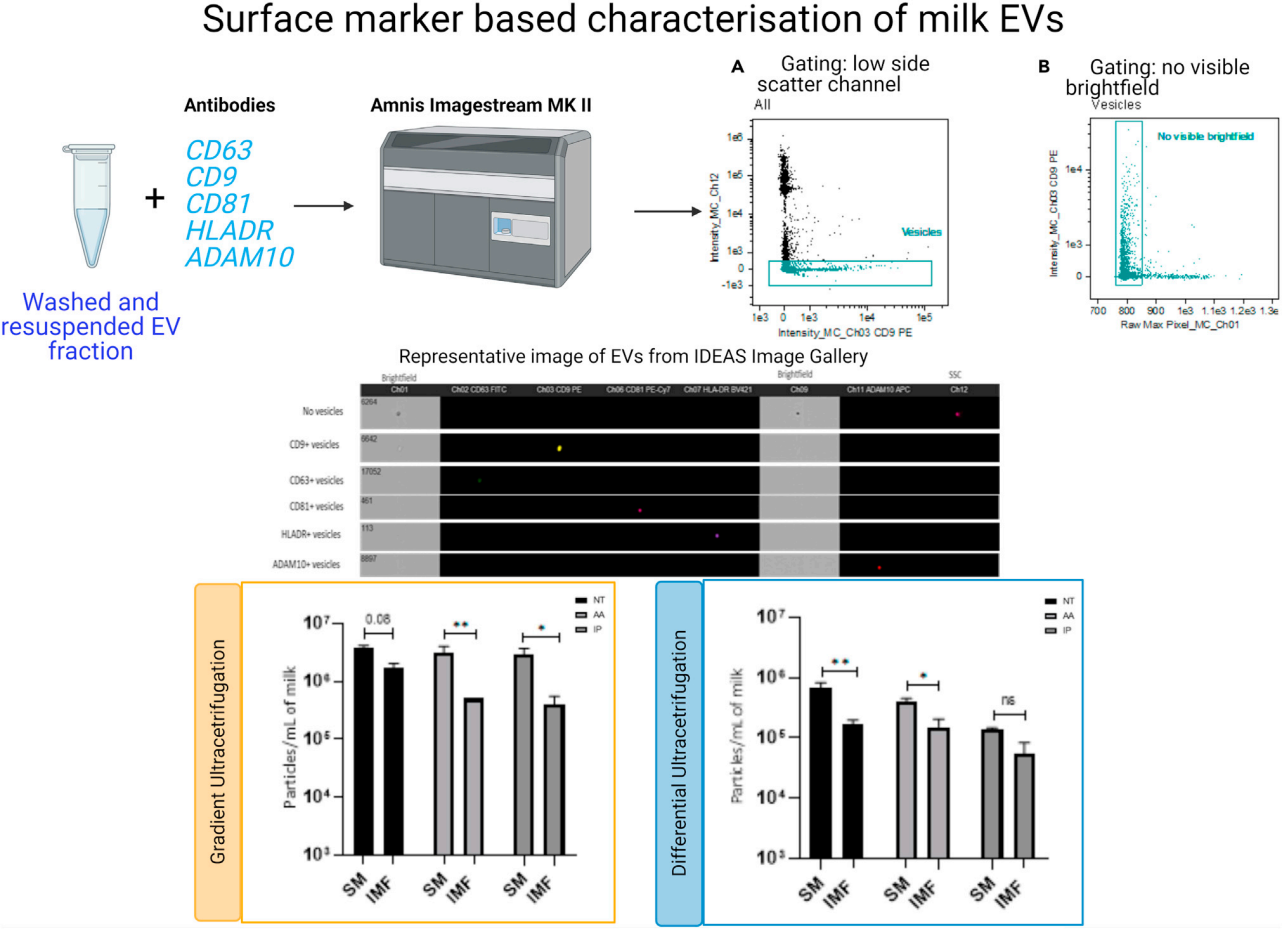
Imaging flow cytometry analysis of EV associated markers Number of positive events (particles) in skim milk (SM) and infant milk formula (IMF) samples analyzed by imaging flow cytometry (IFCM). The events were gated for low side scatter channel (SSC) signals and no signals on brightfield, based on EV size and events positive for these gating was acquired as objects (+EVs) in low SSC. The number of positive CD9, CD63, CD81, ADAM10 and HLADR particles in GUC samples are presented in the lower panel. The IDEAS software was used to analyze the data, data presented as means and individual values ± SEM. A two-way ANOVA test was performed to compare differences in EVs in SM and IMF samples from each treatment groups; ∗indicates p<0.05. ∗∗indicates p<0.01 and ∗∗∗indicates p<0.001. Figure reprinted with permission from Mukhopadhya et al. (2021).

**Imaging flow cytometry analysis:** The analysis of intact EVs based on the presence of EV surface markers CD9, CD63, CD81, ADAM10 and HLADR was performed using imaging flow cytometry (IFCM) and the gating strategy, representative images and observed data is presented in Figure 10. The data from crude samples generated by post-DUC are not presented here but have been reported in Mukhopadhya et al. (2021). In post-GUC samples, significant differences in the +EVs/mL of sample for EV-specific markers (CD63, CD9, CD81, ADAM10 and HLADR) were observed, and where there was a difference in the surface marker, the levels were always higher with the SM EV compared to the IMF EVs. It is also interesting to note that there were no differences observed between non-treated vs. acid-treated samples. This indicates that although there are casein micelles present in these milk EV fractions, the antibodies used for our IFCM analysis only bind to the EVs without non-specific binding. Hence, only the EVs are detected by IFCM and micelles and protein aggregates are undetected by this technique (unlike NTA which accesses particles but cannot decipher EVs from other particles).

## Limitations

Comparing the centrifugations steps: while the GUC itself requires approximately 18 h for centrifugation, the DUC requires approximately only 6.25 h. DUC can accommodate a larger volume of samples (38 mL) compared to GUC, which can only accommodate 2.3 mL per sample. However, it is interesting to note that although DUC is a quicker technique, samples generated post-GUC were more suitable for downstream analysis.

For GUC, the pellets generated post IP and AA treatment of SM and GUC were characterized by all the techniques and were highly suitable, whereas the pellets generated post-NT was not suitable to be analyzed by IFCM as a high number of casein micelles may lead to clogging of the capillary tubes. Compared to SM, IMF pellets generated by GUC post IP and AA were also suitable for all characterization techniques, although very few and disrupted EVs were identified. No intact EV like structures were identified with TEM in IMF_IP samples. In both NT samples from SM and IMF, generated post-GUC, one limitation seems to be the high amount of milk proteins present. Since these proteins, especially caseins, tend to form micelles, they are not the best samples to use in techniques that use light diffraction. On contrary, AA and IP treatment are highly suitable, as they efficiently remove milk proteins, thus increasing the chance to separate pure EVs. However, immunoblotting seems to be the best technique for all the samples, as the binding of the antibodies to the EV markers are specific and reliable data is generated from this technique.

For DUC generated pellets, for both SM and IMF, the major limitation is the presence of a high amount of contaminating proteins, such as caseins. Especially for the NT samples, the presence of casein and whey milk proteins was confirmed in every pellet collected after each centrifugation step, rendering most of these samples unsuitable for further characterization, apart from immunoblotting. Although the AA and IP treatments removed milk proteins, visualized by SDS-PAGE, the characterization of the pellets generated post-DUC was difficult. For instance, with TEM, a high amount of debris was observed with most of the samples, making it difficult to identify EV structures. Similarly, using these samples for flow cytometric analysis was difficult and only the 200 K samples were analyzed. Again, as mentioned above, immunoblotting is the technique of choice here as this technique helps in identifying the presence of EV markers efficiently. Although, one point of concern is the trapping of EVs within the casein micelles and getting precipitated at a lower ultracentrifugation speed compared to the actual speed where EVs should be precipitated.

## Troubleshooting

### Problem 1

Milk preparation issues (step 1)

### Potential solution

If using liquid milk, users should ensure that any free fat, milk solids, protein aggregates are removed from the milk before proceeding with EV separation. If using raw milk, we have detailed additional steps to be followed in step 1.

In case raw milk is being stored for further EV separation, the defatting and removal of milk solids, protein aggregates and free cells should be performed before storage of milk at −80°C.

Spray-dried milk can be used, as demonstrated in the current protocol. However, as the manufacturer’s recommendations to reconstitute spray-dried milk vary, users should ensure that the milk powder is dissolved and then proceed with the protocol.

### Problem 2

Sufficient casein removal (step 5 or 9)

### Potential solution

We have demonstrated that the separation of highly enriched EV fractions from milk involved casein protein removal. Insufficient removal of casein may lead to blockage of filters (step 5 or 9) or during downstream analysis. Hence, care should be taken during the centrifugation step and filtration step post-acid treatment to ensure that the casein pellet is not disturbed.

It is also essential to keep the pH as close as possible to 4.6. In case the pH is higher than 4.6, it will not precipitate the casein completely whereas if the pH is highly acidic, this may affect the EVs.

### Problem 3

Low yield of EVs (steps 4 to 9)

### Potential solution

Milk should be brought to room temperature before applying this protocol. Using frozen or hot milk may lead to the formation of crystals or foam, respectively, further hindering the filtration process (steps 5 or 9).

It is essential to bring the pH of the milk to 4.6 for efficient precipitation of casein. Issues with pH meter or filtration steps may lead to contamination of milk whey with casein (steps 4 or 7).

Collection of 1 mL fraction post-GUC should be performed with utmost care to not disturb layers in order to not lose the EV enriched fractions.

### Problem 4

Collapsing of tubes during ultracentrifugation (steps 18 and 28)

### Potential solution

For steps 18 and 28, the ultracentrifuge related instructions should be strictly followed. In case tubes are not filled up to the maximum volume, there is a higher chance of tubes collapsing leading to loss of samples as well as negatively affecting the ultracentrifuge rotor.

### Problem 5

Choice of techniques to quantify EV concentration (steps for post-separation quantification)

### Potential solution

Based on the data presented in Table 1, it may seem that there is a loss of EVs in the acid-treated sample compared to non-treated milk samples. However, this reflects the limitations of the technique (nanoparticle tracking analysis (NTA)) rather than the actual loss of EVs. While NTA technique is highly preferred and frequently used technique to characterize particle concentrations within a sample, it does not differentiate between EVs, protein aggregates, or any other particle type (> approximately 30 nm) that may be present. Hence, complementary characterization techniques should be used to fully characterize the samples. Interestingly, we do see a decrease in EV concentration between non-treated vs. acid-treated samples, this reflects the removal of casein micelles (Figure 8B) rather than loss of EVs.

## Resource availability

### Lead contact

Further information and requests for resources and reagents should be directed to and will be fulfilled by the lead contact, Lorraine O’Driscoll (lodrisc@tcd.ie).

### Materials availability

This study did not generate new unique reagents.

## Data Availability

The protocol did not generate new unique code.
